# Recent Advances in Synthesis and Applications of Calixarene Derivatives Endowed with Anticancer Activity

**DOI:** 10.3390/molecules29174240

**Published:** 2024-09-06

**Authors:** Elżbieta Wojaczyńska, Marta Ostrowska, Małgorzata Lower, Natalia Czyżyk, Anna Jakieła, Alberto Marra

**Affiliations:** 1 Faculty of Chemistry, Wrocław University of Science and Technology, Wybrzeże Wyspiańskiego 27, 50-370 Wroclaw, Poland; elzbieta.wojaczynska@pwr.edu.pl (E.W.);; 2 Clinical Department of Clinical Oncology, 4th Military Clinical Hospital with Polyclinic in Wrocław, R. Weigla 5, 50-981 Wroclaw, Poland; annajakiela1@gmail.com; 3 Institut des Biomolécules Max Mousseron (IBMM), Université de Montpellier, 1919 Route de Mende, CEDEX 5, 34293 Montpellier, France

**Keywords:** azacalixarenes, calix[4]arenes, calix[4]resorcinols, calixpyrroles, chemotherapy, dihomooxacalixarenes, pillarenes, thiacalixarenes

## Abstract

Calix[n]arenes, macrocycles constituted of 4–8 phenol moieties linked through methylene bridges, are stable molecules that can be selectively functionalised at the upper or lower rim. It has already been demonstrated that calixarene derivatives can be biologically or pharmacologically active compounds. More recently, suitably functionalised calixarenes and calixarene analogues (dihomooxacalixarenes, thiacalixarenes, calix[4]resorcinols, azacalixarenes, calixpyrroles, and pillarenes) were found to act as anticancer agents, at least in in vitro assays. We are reporting on the latest progress in this research field.

## 1. Introduction

Among the structurally well-defined multivalent platforms (e.g., dendrimers, cyclodextrins, fullerenes, and cyclopeptides), calixarenes are of particular interest due to their chemical stability, the adoption of various fixed conformations, and selective functionalization at either the lower or upper rim or both [1,2,3,4,5,6]. Although different families of calix[n]arenes are known, the calix[4]arenes, macrocycles constituted of four phenol units connected through methylene bridges, have been more often employed as scaffolds because they are easily synthesised [7], and their conformations can be controlled by suitable substituents at the lower rim. In fact, unmodified calix[4]arenes are conformational mobile macrocycles which adopt a cone conformation because of the cooperative hydrogen bonds between hydroxy groups. However, the addition of short carbon chains at the lower rim (e.g., by *O*-propylation of the four phenolic hydroxyls) inhibits the oxygen-through-the-annulus rotation, leading to the isolation of the blocked conformational isomers (cone, partial cone, 1,2-alternate, and 1,3-alternate) [8].

Calixarene-based multivalent constructs featuring new biological or pharmacological properties [9,10,11,12,13] have been extensively exploited in the glycobiology [14], gene delivery [15], controlled drug release [16], and antimicrobial medicine [17,18] fields. More recently, this multivalent platform has also been employed to prepare potential anticancer drugs, as proved by the publication of numerous articles which have been in part reviewed [19,20,21,22,23,24,25]. The calixarenes, a long time ago considered just as cyclo-oligomeric by-products of the phenol-formaldehyde condensation to produce Bakelite [1], are now on the front line against cancer, a leading cause of death worldwide (10 million in 2020), in general, originated by widespread metastases [26]. It is well-known that in order to fight cancer, developed each year by 400,000 children, prevention and early detection are essential to reduce mortality; nevertheless, the discovery of efficient and less toxic drugs is of the utmost importance. In the present review, covering the articles published over the last six years (2019–2024), we describe the structures as well as the in vitro and in vivo pharmacological properties of mono- and multivalent architectures assembled onto a calix[n]arene platform or onto less common macrocyclic scaffolds such as dihomooxacalixarenes, thiacalixarenes, calixresorcinols, azacalixarenes, calixpyrroles, and pillarenes. More specifically, the covalently linked calix[n]arene derivatives are described in Section 2, while the calix[n]arene-based complexes are presented in Section 3. Finally, all the analogues of calixarenes (dihomooxacalixarenes, thiacalixarenes, calixresorcinols, azacalixarenes, calixpyrroles, pillarenes) are described in Section 4.

## 2. Calixarene-Based Anticancer Compounds

### 2.1. Spiro-Indoline Calixarenes

**Figure 1:**
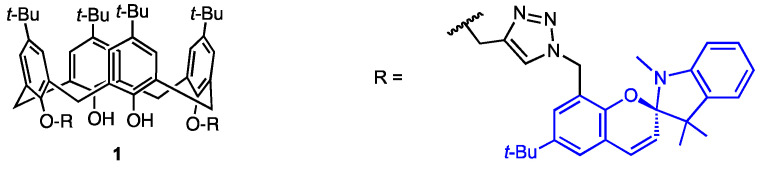
Spiro-indoline-conjugated calix[4]arene.

Rao and co-workers [27] described in 2019 the preparation of the racemic 1,3-di-spiro-indoline calix[4]arene derivative **1** (Figure 1), whose copper(II) complex was found as a potential anticancer agent. The Cu-mediated azide-alkyne cycloaddition (CuAAC) between di-*O*-propargyl-calixarene and a trisubstituted benzyl azide gave a bis-triazole derivative, which was reacted with 1,2,3,3-tetramethyl-*3H*-indolium iodide to afford a bis(spiro) calix[4]arene-based ligand. Its complexation with copper(II) perchlorate led to a 1:1 complex, as proved by spectroscopic analyses (UV-Vis, NMR, EPR, IR) and fluorimetric studies. The DFT calculations indicated a distorted tetrahedral geometry with phenolic oxygen atoms and triazole nitrogen atoms engaged in coordination. The Methyl Thiazolyl Tetrazolium (MTT) assay performed with MDA-MB-231 cancer cells revealed that, in contrast to the ligand itself, the copper(II) complex exhibited a high antiproliferative potency with an IC_50_ value of 165 nM. Its toxicity for the reference line was, however, not checked in the study. The cell-cycle analysis was performed and revealed the arrest of the SubG1 population. Cell imaging studies showed that the ligand accumulated in the mitochondrial region of the cells, as demonstrated by its red fluorescence.

### 2.2. Proline Functionalised Calixarenes

**Figure 2:**
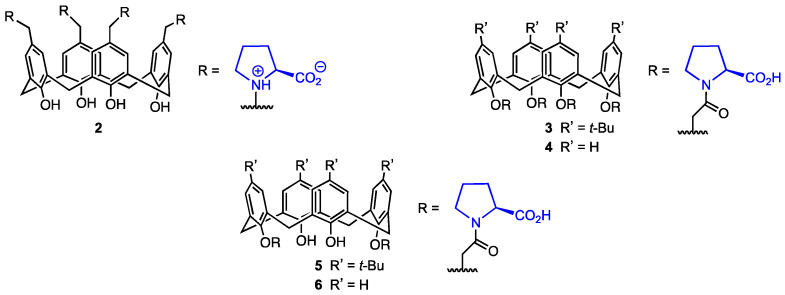
L-proline calix[4]arene derivatives.

In 2020, Karakurt and co-workers [28] synthesised a series of upper- and lower-rim calix[4]arene derivatives functionalised with L-proline (**2**–**6**, Figure 2) and evaluated their activity and cytotoxicity. Increased levels of proline were proven to delay cell aging [29], and chiral proline-modified calix[4]arenes have shown enantioselective inhibition of the formation of L1-p, a pentamer essential in the human papillomavirus (HPV) virion particle assembly [30]. Since HPV is one of the causes of human cervical cancer [31], this makes proline-modified calixarenes an interesting perspective in cancer prevention, and their antiviral properties could be used in drug development. However, it has been unclear so far whether they display significant levels of activity against already existing cancer cells.

The L-proline-based calixarene derivatives **2**–**6** (Figure 2) were evaluated for their activity against A-549, PC-3 (prostate cancer), DLD-1 (colon cancer), HePG2 (liver cancer) cell lines, and PNT1A prostate healthy cell lines by treating the cells with various calixarenes. The cytotoxicity was determined using the Alamar Blue assay. The calixarene **6** containing two proline units at the lower rim was found to have the highest cytotoxicity against DLD-1 and HEPG2 cells (IC_50_ = 29.25 and 64.65 μM), but it displayed high toxicity against the healthy cell line PNT1A (65.91 μM). The *tert*-butylated analogue **5** showed high toxicity against A549 (15.70 μM) and PC-3 (23.38 μM) cells; however, its cytotoxicity against healthy cells was the highest of all the investigated derivatives (40.06 μM). It could be argued that although the *N*-acyl-proline derivatives are active against cancer cells, their cytotoxicity may hinder their usage as selective drugs in antitumour therapy. On the other hand, compound **2**, bearing L-proline moieties in a zwitterion form, did not display cytotoxicity against PNT1A cells (IC_50_ > 200 μM) while still showing relatively high activity against DLD-1 cells (43.00 μM). Although **2** was not particularly efficient against other cell lines, it could potentially be used in colon cancer therapy because of its activity against DLD-1. Finally, calixarenes **3** and **4** displayed higher cytotoxicity against the healthy cell lines than against A549, PC-3, and DLD-1. Compounds **2**–**6** significantly increased the apoptotic cell death. The high activity of the calixarene containing zwitterionic proline against DLD-1 cells may lie in the genetic differences between the cells. In DLD-1 cells, the genes that code enzymes crucial to the proliferation of the cells are mutated, so their expression is much lower than it normally would be in healthy cells. Therefore, the L-proline calixarenes displayed cytotoxic behaviour against various human cancer cell lines, but their selectivity needs improvement. Comparing the cytotoxicity of the L-proline-substituted calixarenes with their unsubstituted counterparts might also allow for assessing the importance of L-proline in the antitumour activity of the calixarene derivatives. It should also be mentioned that although proline itself proved to postpone cell aging, its metabolism is suspected to at least somewhat sustain cancer cell proliferation, survival, and spread. Pyrroline-5-carboxylate reductase (which catalyses the last step in proline biosynthesis) and proline dehydrogenase (which catalyses the first step of proline catabolism) have been associated with the progression of several malignancies [32]. Additionally, there is evidence that cancers utilise amino acids to support their metabolism, with proline being used for ATP generation specifically [33] and proline levels being limiting for protein synthesis in some tumours [34]. On the one hand, some metabolic proline enzymes work to promote cell death, but on the other hand, they may act as oncogenic proteins. Proline metabolism and the enzymes that have a key role in it have been exposed as targets for potential anticancer drug development themselves [32]. Due to the complex interactions of proline and the influence of its metabolism on cancer cell proliferation, the L-proline-based calixarene derivatives may not prove to afford the expected results in terms of antitumour activity.

**Figure 3:**
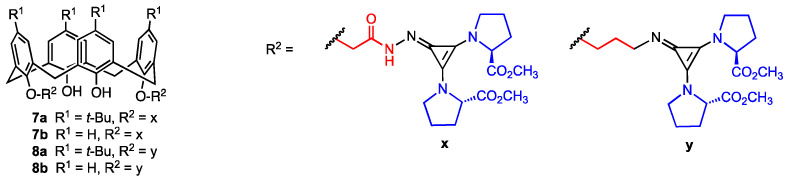
Calix[4]arenes functionalised with cyclopropenimine and L-prolinate units.

The four tetraprolinate calix[4]arene derivatives **7a**,**b** and **8a**,**b** (Figure 3) equipped with two “superbase” cyclopropenimine moieties at the lower rim were described by Yilmaz and co-workers in 2022 [35]. The compounds **7a**,**b** were synthesised from *p*-tert-butylcalix[4]arene and calix[4]arene, respectively, through conversion of the starting compounds into diesters by reaction with bromomethyl acetate. For the synthesis of calix[4]arenes **8a**,**b**, treatment with *N*-(3-bromopropyl)phthalimide yielded the corresponding bis(phthalimidopropoxy) derivatives. In the second step, the four precursors were reacted with hydrazine to obtain bis-hydrazide or bis-amino-calixarenes, respectively. In the final step, the addition of dimethyl(3 chlorocycloprop-1-ene-1,2-diyl)di-L-prolinate led to the target compounds **7a**,**b** and **8a**,**b** in 55–75% yield. In vitro assay conducted on five human cancer lines (DLD-1, A-549, HEPG2, PC-3, PNT1A) revealed that these compounds exhibit antiproliferative activity with IC_50_ values ranging from 4.7 to 288.3 μM. Notably, the most active compound (**8a**) demonstrated significantly higher cytotoxicity against DLD-1 (human colon cancer) and PC-3 (human prostate cancer) compared to the normal human epithelial cell line PNT1A, showing greater promise than simple calixarene-proline derivatives. However, a tendency of these superbase compounds to decompose in solution was also observed, which could pose significant challenges to their stability and practical application.

### 2.3. Aspartic and Glutamic Acid-Functionalised Calix[4]arene Derivatives

**Figure 4:**
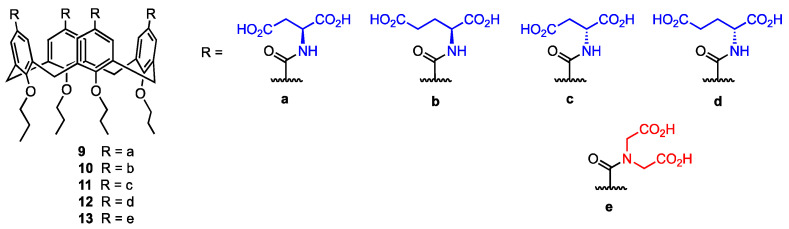
Amides of calix[4]arenes with aspartic, glutamic, and 2,2′-azanediyldiacetic acids.

Mocerino and co-workers [36] focused on the ability of acid-functionalised calix[4]arenes to inhibit human papillomavirus (HPV) pentamer formation. Inhibiting the self-assembly of the L1 virus monomer into a pentamer, which then forms the capsid, would target the viral infection itself, achieving substantial progress in the development of HPV infection treatment by preventing the spread of the virus. Calixarenes functionalised with acidic groups can interact with lysine and arginine in the L1 protein, inhibiting pentamer formation [37]. In this study, carboxylic acid calix[4]arenes functionalised with L- and D-aspartic and glutamic acid **9**–**12** were used (Figure 4).

The inhibition of HPV L1 pentamer formation was determined via size-exclusion chromatography for various concentrations of the α-amino acid-functionalised calix[4]arenes **9**–**12**, as well as the 2,2′-azanediyldiacetic acid (usually called iminodiacetic acid) calix[4]arene **13**. The L-configured compounds **9** and **10** displayed the best inhibitory properties, achieving IC_50_ values of 0.72 mM for **9** and 0.76 mM for **10**. The D-amino acid derivatives **11** and **12** appeared to be less active in their inhibition of L1 pentamer formation, with IC_50_ values of 1.87 mM for the D-aspartic acid calixarene **11** and 2.67 mM for the D-glutamic acid derivative **12**. The 2,2′-azanediyldiacetic acid calix[4]arene **13** showed no inhibition of the pentamer formation at a ratio of 300:1 (**13**:GST 1), which is most likely linked to the restricted conformational flexibility of the acidic group compared to the compounds bearing aspartic and glutamic acid. Moreover, **13** is a tertiary amide, while the other compounds are secondary amides able to form hydrogen bonds. Therefore, the high potential of calixarenes **9**–**12** in the inhibition of the L1 pentamer formation can be explained by the flexibility of the amino acid side chain and the presence of the hydrogen of the secondary amide, which in turn allows the binding to the positively charged sites on the L1 protein surface. These derivatives could be further used to develop inhibiting agents able to prevent the HPV L1 pentamer formation, targeting the viral infection in its initial phase.

### 2.4. Amide Functionalised Calix[4]arene Derivatives

**Figure 5:**
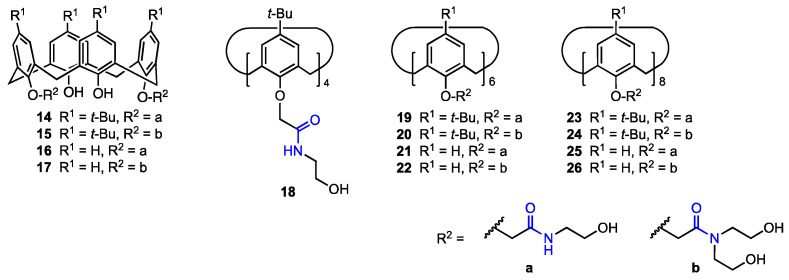
Calix[n]arenes functionalised with ethanolamine or diethanolamine moieties.

In 2016, Yan and co-workers [38] developed a route toward calix[n]arenes (n = 4, 6, 8) bearing multiple ethanolamine or diethanolamine units at the lower rim (**14**–**26**, Figure 5). Five calix[4]arene, four calix[6]arene, and four calix[8]arene derivatives were prepared and characterised by spectroscopic analyses, with the X-ray structure of one calix[4]arene derivative also obtained. Preliminary cytotoxicity assays performed on six cancer cell lines (A549, SKOV3, SW1990, Hela, Raji, MDA-MB-231) indicated that the larger macrocycles, i.e., the calix[6]arene and calix[8]arene derivatives, were generally inactive, likely due to their inability to permeate the cell membrane. Among the calix[4]arenes, those bearing *tert*-butyl groups at the upper rim proved to be more effective, showing IC_50_ values in the low μM range for all tested cell lines. Further studies on human ovarian carcinoma cells (SKOV3) revealed that these compounds induce cell apoptosis through the up-regulation of the Caspase 3 and p53 proteins.

**Figure 6:**
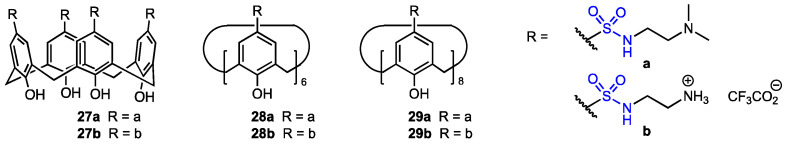
Calix[4]arene, calix[6]arene, and calix[8]arene sulfonamide derivatives.

Water-soluble calix[4]arene, calix[6]arene, and calix[8]arene sulfonamide analogues (Figure 6) were prepared by Bayrakci and co-workers [39] as potential anticancer agents. They were obtained from the corresponding tetra-, hexa- or octa-*p-tert*-butylcalix[n]arenes (n = 4, 6, 8) by reaction with chlorosulfonic acid, and the resulting chlorosulfonated derivatives were treated with either *N*,*N*′-dimethylethylenediamine or *N*-Boc-ethylenediamine. Boc-protected sulfonamides were then converted into quaternary ammonium salts. The structures and cone conformations of the new calixarenes were confirmed by NMR analysis, while the FT-IR spectra proved the presence of the sulfonamide group. All the six calixarene derivatives were moderately cytotoxic toward MCF-7 and MIA PaCa-2 cancer cell lines, with compound **29a** exhibiting the best selectivity for malignant cells (epithelial cell line HEK293 was used as a reference).

**Figure 7:**
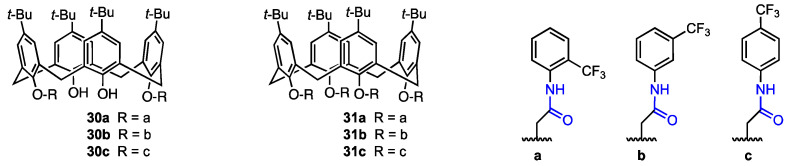
Di- and tetra-amide derivatives of *p*-*tert*-butyl calix[4]arenes synthesised by Oguz [40].

In 2022, Oguz [40] described a series of *p*-*tert-*butylcalixarenes with trifluoromethyl aniline groups as potential anticancer agents. The trifluoromethyl moiety can improve pharmacokinetic properties of organic compounds, such as binding affinity and membrane permeability [41]. The *p*-*tert-*butylcalix[4]arenes were functionalised with trifluoroanilines to obtain di-amide (**30a**–**c**) and tetra-amide (**31a**–**c**) derivatives (Figure 7). Their cytotoxicity against MCF-7 (breast cancer), A549 (lung cancer), HeLa (cervical cancer), HEp-2 (containing HeLa marker chromosomes), and Vero (healthy kidney epithelial cells) cell lines was assessed using the Alamar Blue assay and compared with the cytotoxicity of 5-fluorouracil (5-FU), a well-known clinical antitumoural agent [42].

The 2-(trifluoromethyl)aniline diamide derivative **30a** displayed the highest antiproliferative activity against MCF-7 and A549 cell lines, activity higher than that of 5-fluorouracil against those particular cell lines (IC_50_ = 19.47 μM against A549 and 8.33 μM against MCF-7, compared to 5-FU’s 38.42 μM and 20.61 μM, respectively). On the other hand, the diamide derivative containing a 4-(trifluoromethyl)aniline moiety (**30c**) displayed the highest antiproliferative activity against HeLa cells (IC_50_ = 18.14 μM, compared to 23.31 μM for 5-FU). The 3-(trifluoromethyl)aniline diamide derivative **30b** also displayed some levels of activity, but they were lower than those of **30a**, **30c** and 5-FU. When it comes to the tetra-amide derivatives, the highest activity was also observed for the 2-(trifluoromethyl)aniline derivative **31a** (MCF-7 cells: IC_50_ = 16.16 μM) and the 4-(trifluoromethyl)aniline derivative **31c** (HeLa cells: IC_50_ = 26.80 μM). Once again, the 3-(trifluoromethyl)aniline derivative **31b** did not show particularly high activity levels. Therefore, calix[4]arenes functionalised with trifluoromethylanilines have high antiproliferative activity against certain human cancerous cell lines; however, their highest activity was against HeLa and MCF-7 cells, depending on the structure of the compound, and none of the compounds were active against Hep-2. Additionally, the compounds did not display cytotoxicity against Vero cells, the IC_50_ values being equal to or higher than 197.96 μM (compared to 22.17 μM for 5-FU). It can also be observed that diamide derivatives obtained from the same trifluoromethylaniline isomers were more active than their tetra-amide counterparts, while their cytotoxicity did not increase substantially. Additionally, functionalisation with a 2-(trifluoromethyl)aniline moiety is the most promising synthesis route, as both 2-(trifluoromethyl)aniline derivatives **30a** and **31a** displayed high levels of activity and low cytotoxicity. On the other hand, functionalisation with 3-(trifluoromethyl)aniline did not yield particularly active derivatives. This is likely due to the *ortho* effect in aniline—the *ortho*-substituted compounds are less basic than other isomers. The experimental p*K*_a_ value of 3-(trifluoromethyl)aniline is 3.49 [43] and 2.75 for 4-(trifluoromethyl)aniline [43], while the predicted p*K_a_* for 2-(trifluoromethyl)aniline is 1.10 [44]. It has already been shown that acidic conditions (pH 6.0 and lower) can induce cell death of breast cancer cells [45], so there might be a possibility that the *ortho*-substituted aniline derivatives are more active due to this effect.

**Figure 8:**
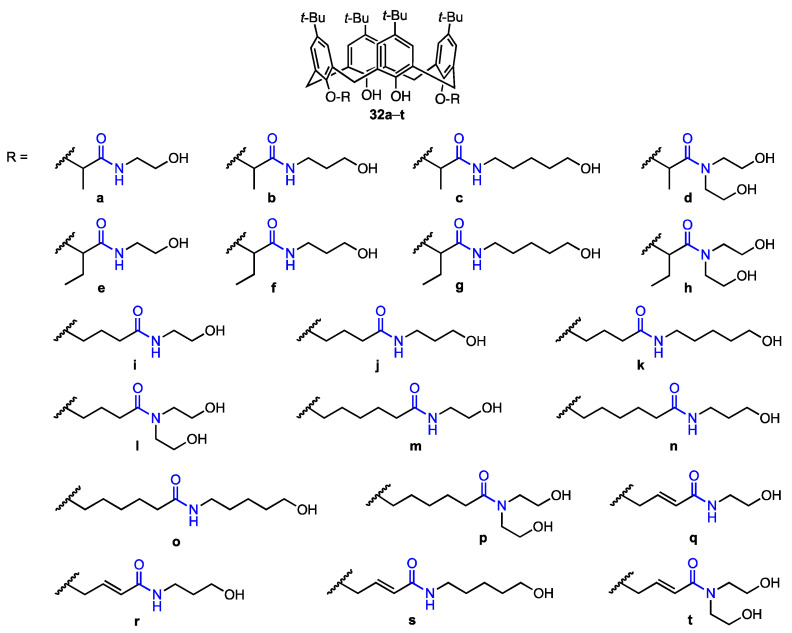
Series of calix[4]arene amides prepared by An, Zheng, and their co-workers [46].

An, Zheng, and their co-workers [46] synthesised twenty calix[4]arene-based carbonyl amide derivatives (**32a**–**t**, Figure 8) and studied their anticancer activity against A549, MCF-7, MDA-MB-231 (breast cancer), HT29 (colon carcinoma), and HepG2 (hepatocellular carcinoma) human cancer cell lines, as well as cytotoxicity against HUVEC cells. Some derivatives were found to be active against all five cancer cell lines (MTT assay). In particular, compound **32h** displayed inhibition rates of 90.4%, 64.2%, 89.6%, 88.2%, and 90.7% against MCF-7, HT29, HepG2, MDA-MB-231, and A549, respectively, at 10 μM (5 h), and also showed good resistance against HUVEC cells (9.6% inhibition rate). Compared to the previously published [38] calix[4]arene **14** (see Figure 5) bearing simple *N*-hydroxyethyl-amide groups, **32h** displayed activity that was 3.2 times higher against A549 and 6.8 times higher against MDA-MB-231. Based on the inhibitory activity of other derivatives, it was determined that as the length of the alkyl chain between the oxygen atom in the phenolic hydroxy group and amide at the lower rim increases, the activity decreases drastically, and as the steric hindrance between the lower phenolic hydroxyl group and amide increases, the activity also increases. Since compound **32h** showed low cytotoxicity against healthy human cells while displaying IC_50_ values of 1.20 μM (A549) and 0.66 μM (MDA-MB-231), it appears to be the most promising anticancer drug candidate. The mechanism of inhibition was further determined—it was shown in a wound-healing assay that the derivative hinders cell migration of MDA-MB-231 (with the cell migration being prevented to a higher extent as the concentration increases). Apoptosis was also investigated, as the MDA-MB-231 cells were stained with Hoechst 33342, and the cells displayed signs of apoptosis under various concentrations of **32h**, thus suggesting that it has a pro-apoptotic effect. This activity was investigated by flow cytometric analysis, which indicated that the addition of the compound may induce cell death and block the G0/G1 phase of the cell cycle. The calixarene derivative induced the activation of caspase-3 (an executive molecule in apoptosis regulation) in the MDA-MB-231 cells, with the concentration of activated caspase-3 increasing as the concentration of the derivative increased. Additionally, the presence of **32h** induced the expression of Bax protein, which is pro-apoptotic, and hindered the expression of Bcl-2 protein (anti-apoptotic). It can thus be concluded that **32h** is a potential antitumour drug, since it has a high anti-proliferative ability and can possibly induce apoptosis in the cancer cells, not just inhibit proliferation.

### 2.5. Fluorescent Lower Rim-Functionalised Calix[4]arene Derivatives

**Figure 9:**
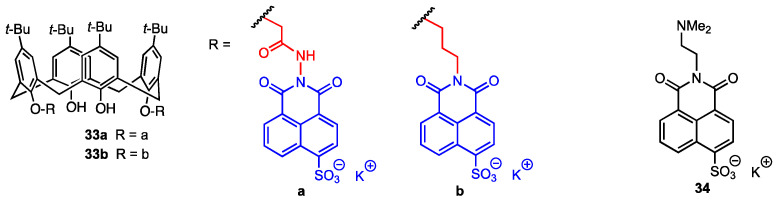
Fluorescent 4-sulfo-1,8-naphtalimide derivatives of *p*-*tert*-butylcalix[4]arene.

Yilmaz and co-workers [47] synthesised two fluorescent calix[4]arenes bearing 4-sulfo-1,8-naphtalimide moieties linked at the lower rim through hydrazide (**33a**) or aminopropyl (**33b**) chains (Figure 9). It has already been proven that calix[4]arenes are effective against human ovarian carcinoma cells [48], and 1,8-naphthalimide derivatives can be used not only as supramolecular building blocks and smart materials but also as biological probes [49,50] and active compounds against breast, cervical, prostate, and epidermal carcinoma cells [51,52,53]. The calixarenes **33a** and **33b** were tested for their activity using a DLD-1 colorectal adenocarcinoma cell line and healthy colon epithelial cells (CCD-18Co). IC_50_ was found to be 12.95 μM for **33a** and 16.13 μM for **33b** against the DLD-1 cell line. On the other hand, for the healthy cell line, the IC_50_ values were 508 μM (**33a**) and 269 μM (**33b**), which means that the proliferation of the normal cells was not affected by the calixarene derivatives. At the same time, it was assessed that the mechanism of action of the compounds was cytotoxic, not cytostatic, as the number of cells was reduced after treatment. Additionally, the activity of *N*,*N*-dimethylaminoethyl-4-sulfo-1,8-naphthalimide **34** was evaluated against the DLD-1 cell line, and no significant effect was observed. Therefore, the increase in activity when the naphthylimide is bound to the macrocyclic scaffold indicates that the calix[4]arene plays a vital role in cell death.

### 2.6. Quaternary Ammonium-Modified Azocalix[4]arene

**Figure 10:**
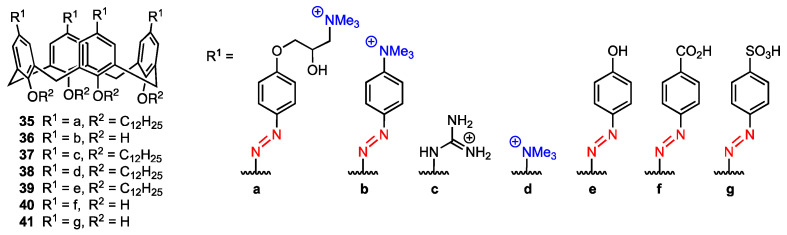
Series of calixarene-based immunogenic cell death inducers.

Shi, Guo, Liu, and co-workers [54] reported the synthesis and properties of the quaternary ammonium salt azocalix[4]arene **35** (Figure 10) that was proved to drive immunogenic cell death (ICD) and could potentially be used in cancer immunotherapy, as well as be used alongside anticancer drugs to improve their performance via host–guest bonding. The other analogues (**36**–**41**, Figure 10) were synthesised to assess the impact of the structure of the compound on ICD-inducing activity.

All compounds were exposed to mouse breast cancer cells (4T1) and incubated for 12 h using the anticancer paclitaxel (PTX) as a positive control and phosphate buffer saline (PBS) as a negative control. The activity was assessed as the ratio of surface calreticulin (CRT) positive cells, as surface-exposed pre-apoptotic CRT is one of the typical damage-associated molecular patterns connected with ICD inducing. The highest ratio of CRT-positive cells was achieved when treating the cells with compound **36** (17.7% compared to 2.55% when treating the cells with PTX), and other derivatives provided a significant ratio as well (2.99% for **35**, 2.96% for **38**). The data suggests that the presence of positively charged groups at the upper rim of the calixarene provides a higher ICD-inducing activity. Additionally, in the case of **36**, the quaternary ammonium group is directly linked to the azobenzene group, and the conjugated system of the molecule may be the reason for its high activity. To confirm the activity of the compounds, the exodus of high mobility group box 1 (HMGB-1) of cells after treating them with calixarenes and PTX was investigated. It was found that only cells treated with **36** and PTX displayed low levels of intracellular HMGB-1 and high levels of extracellular HMGB-1, which indicates an exodus of HMGB-1 after the treatment. As for the mechanism of the ICD-inducing activity, it was determined that the calixarenes **35**, **36**, and **38** promoted endoplasmic reticulum stress (assessed by pEIF2α levels in the cells), with the conclusion being that all those three derivatives induce ICD by promoting ER stress and EIF2α phosphorylation (a known mechanism for ICD inducers [55]). Using time-dependent cellular uptake analysis, it was determined that the amphiphilic derivatives **35** and **36** featured faster cellular uptake kinetics. This suggests that besides the presence of the positively charged groups at the upper rim and the structure of the calixarene skeleton itself, the way the compounds assemble into aggregates in aqueous media will also play a role in cellular uptake efficiency (and ICD-inducing activity). Due to its macrocyclic structure, **36** can bind to anticancer drugs and could potentially be used with chemotherapeutics to improve their performance. To further investigate this possibility, four anticancer drugs not able to induce ICD, i.e., methotrexate (MTX), chlorambucil (Chl), etoposide (ETO), and pemetrexed (PEM) were loaded into **36**, and their capability to activate immune responses connected with ICD was assessed. Cells treated with only the anticancer drugs, as well as complexes of the drugs and **35** or **40**, did not cause surface CRT exposure, while cells treated with the complexes of **36** and the anticancer drugs showed significant CRT exposure on the cells, meaning that while the drugs themselves are not ICD-inducers, the complexes can act as such. Using the sulfonated aluminium phthalocyanine (AlPcS_4_) fluorescent probe loaded into **36** and treating different biological species with the complex, it was also determined that AlPcS_4_ was delivered specifically into tumour tissues. This behaviour indicated that the calixarene can be used to deliver anticancer drugs into tissues in a way that avoids interference with other tissues. The effect of using a **36**-methotrexate complex for cancer treatment was also assessed during in vivo studies using 4T1 tumour-bearing mice. Treatment using the complex was the most effective and significantly improved the survival rate compared to PBS, MTX, and **36** treatments. It was also demonstrated that **36** had low in vivo cytotoxicity, while the enhanced tumour suppression observed with **36**-methotrexate was most likely due to synergy between the compounds, which was further proven by flow cytometric analysis. The results indicated that the calixarene derivative **36** can act not only as a standalone ICD-inducer but also as a drug delivery agent that works synergistically with antitumour drugs.

### 2.7. Calixarenes Bearing Isatin Moieties

**Figure 11:**
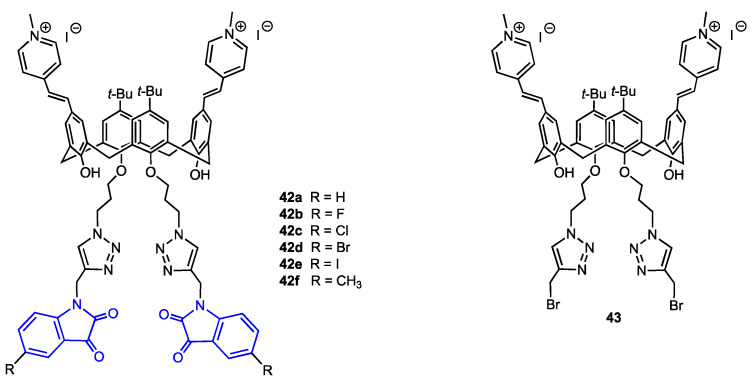
Structures of the calix[4]arenes **42a**–**f** bearing isatin units and the reference calixarene **43**.

Yilmaz and co-workers [56] developed a route to calix[4]arenes bearing two methylpyridinium cations at the upper rim and two isatin moieties attached to the lower rim (Figure 11). These derivatives were targeted to the mitochondria of cancer cells. The synthesis started with bis-formylation of upper rim aromatic rings followed by a reaction with 1,4-dimethylpyridinium iodide. At the lower rim, two azide-functionalised arms were linked to the hydroxyl groups, then, CuAAC reaction with six propargylated isatin derivatives gave the desired compounds **42a**–**f**. The triazole derivative **43** lacking isatin pharmacophore was prepared as well, using propargyl bromide in the last step. The compounds were evaluated for their antiproliferative activity against two types of breast cancer cells, MCF-7 and MDA-MB-231, and a human normal epithelial cell line PNT1A. The observed IC_50_ values were in the 3–20 μM range, in most cases lower than that displayed by 5-fluorouracil used as a reference. The isatin-bearing derivatives were more active in comparison to the compound **43** which, however, exhibited some cytotoxicity due to the presence of 1,2,3-triazole moiety. The toxicity toward the healthy cells was lower for all derivatives, the IC_50_ being 4–18 times smaller than for malignant cells. The confocal microscopic study revealed the localisation of compound **42c** in the mitochondria of MDA-MB-231 cells. This compound was also identified as a good apoptotic agent and a potent inhibitor of aromatase enzyme, which is also a target in breast cancer therapy.

### 2.8. Non-Functionalised Calixarenes

**Figure 12:**
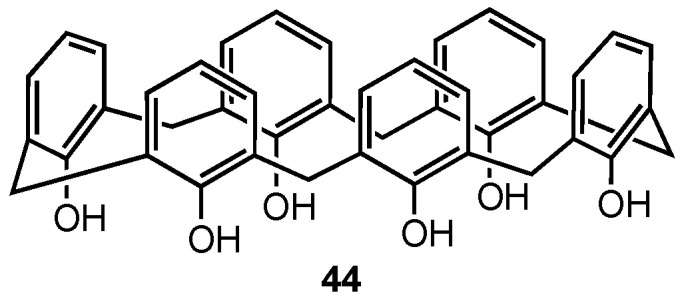
Structure of the calix[6]arene.

Ferreira-Halder and co-workers have been exploring the use of unsubstituted calix[6]arene **44** (Figure 12) for the treatment of pancreatic cancer since 2013, initially reporting that **44** was more potent than both gemcitabine and 5-fluorouracil in reducing the viability of the drug-resistant human pancreas carcinoma Panc-1 cell line [57]. Moreover, calix[6]arene was able to suppress the signal transduction of the Mer and AXL tyrosine kinase receptors, which are overexpressed in this type of cancer. In 2020, they further demonstrated that **44** induced the degradation of the AXL receptor tyrosine kinase in Panc-1 cells via clathrin-mediated endocytosis [58]. Furthermore, they found that **44** could inhibit the migration and invasion of Panc-1 cells by downregulating FAK (a downstream mediator of AXL) activity and reducing the expression of the matrix metalloproteinases MMP-2 and MMP-9, which are responsible for initiating metastasis of cancer cells. In a 2024 study, the same research team evaluated the potential of calix[6]arene **44** not only to decrease the activity of MMP-2 and MMP-9 but also to prevent the release of extracellular vesicles (EVs) [59]. EVs induce metastasis by promoting intercellular communication and enhancing carcinogenesis, making them an interesting target for treatment strategies. Experiments were conducted using two pancreatic cancer cell lines, PANC-1 and MIA PaCa-2. Western-blot analysis revealed that PANC-1 cells exhibited a higher expression of proteins involved in EV secretion and contained a higher concentration of EVs compared to the MIA PaCa-2 cell line. It was found that the calix[6]arene reduced both the viability of pancreatic cells themselves and the release rate of EVs by PANC-1 cells at subtoxic concentrations (5 µM), suggesting that **44** is capable of preventing EV biogenesis. After treatment with a 10 µM solution of **44**, MMP-2 and MMP-9 expression was reduced in both pancreatic cancer cell lines, indicating that calix[6]arene is able to reduce MMP activity but only at high concentrations that may make it not viable for human treatment. Nevertheless, the ability of calix[6]arene **44** to prevent EV production makes it a promising candidate for alternative cancer treatments.

## 3. Complexes of Calixarene as Potential Anticancer Drugs

### 3.1. Complex of Calix[4]arene Tetramalonate with Cis-Diammonia-Platinum(II)

**Figure 13:**
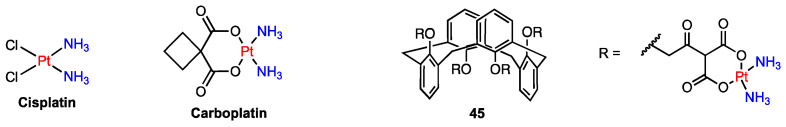
Platinum complex of a calix[4]arene tetramalonate.

Cisplatin and carboplatin, despite being well-known anticancer drugs, suffer from short blood circulation times due to their low molecular weight, leading to reduced tumour uptake and limited intracellular binding. To address this issue and aim to increase the half-life of the platinum complexes, Pur and Dilmaghani prepared the water-soluble tetra-platinum(II) complex **45** (Figure 13) from a calix[4]arene in 1,3-alternate conformation functionalised with four malonate moieties [60]. In vitro tests demonstrated that **45** was more cytotoxic against three human carcinoma cell lines (HepG2, MCF7, A549) compared to carboplatin and three other monomeric platinum complexes, suggesting its potential as a more effective anticancer agent.

### 3.2. Complex of Sulfonatocalix[4]arenes with Pyridinium-Tetraphenylethylene

**Figure 14:**
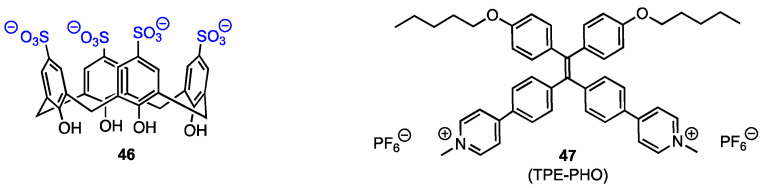
Structures of the calixarene tetra-sulfonate and TPE-PHO.

Feng, Ding, Tang, and their co-workers [61] synthesised the host–guest complex of the water-soluble calixarene **46** (Figure 14) with an aggregation-induced emission luminogen (AIEgen), the pyridinium-functionalised tetraphenyl-ethylene **47** (TPE-PHO). The complex was designed to improve the biocompatibility and selectivity in photodynamic therapy (PDT) under light irradiation, aiming to obtain a promising non-invasive therapeutic for cancer treatment. The AIEgens are used in PDT as alternatives to traditional photosensitisers that generate reactive oxygen species under light irradiation to cause cell apoptosis, as they are non-emissive or weakly emissive in the solution but have a high fluorescence in the aggregate state. The complex with calixarenes should allow for the reduction of their cytotoxicity against healthy cells. Sulfonatocalix[4]arene **46** was used to form a complex with TPE-PHO in order to inhibit its cytotoxicity and fluorescence, the premise being that at the tumour site, the TPE-PHO **47** would be released from the calixarene cavity by displacement with 4,4′-benzidine dihydrochloride (BZD).

The sulfonate groups at the upper rim of the calixarene allow for the bonding with the positively charged **47**, while the hydrophobic nature of the latter (due to its *n*-pentoxy chains) alongside the water solubility of **46** leads to an amphiphilic aggregate. The photoluminescence of **47** and the aggregate was measured, and it was found that the AIEgen was only weakly emissive, whereas the supramolecular aggregate emitted intense fluorescence at 570 nm, meaning that the pyridinium groups of **47** were encapsulated in the cavity of **46**. Upon the addition of BZD, the fluorescence was restored to its original state, indicating the displacement of **47**. The phototheranostic ability of the complex was determined using MTT assays for testing cell viability of HeLa cells incubated with different concentrations of the AIEgen and the aggregate. At 20 μM, the cell viability was 38% when only **47** was used, but it increased to 79.9% after the supramolecular assembly with **46**, clearly indicating that the aggregate was less cytotoxic. Using cell imaging, it was determined that upon displacement by BZD, the AIEgen translocates from cytoplasm to mitochondria and its cytotoxicity and photoactivity are restored. This feature is especially desirable since targeting mitochondria allows for easier cell apoptosis using PDT when the mitochondrial membrane is damaged. The supramolecular aggregate of **46** and **47** can be thus potentially used in targeted photodynamic therapy against cancer cells to inhibit unwanted cytotoxicity of the drug.

### 3.3. Complex of Calixarenes with Betaine

**Figure 15:**
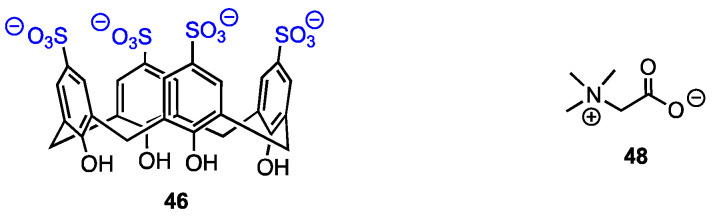
Structures of the calixarene tetra-sulfonate and betaine.

El-Said Azzazy and co-workers [62] reported on an efficient enhancement of the antiproliferative activity of betaine (trimethylglicine, **48**) after complexation with *p*-sulfonatocalix[4]arenes **46** (Figure 15). This work was based on the fact that betaine required high doses to show anticancer action. Its introduction into cancer cells in the form of an inclusion complex opened the possibility of increased concentration; the acidic environment of tumour tissue should result in drug release. The formation of a host–guest complex was confirmed by the upfield shifts of betaine protons observed in the ^1^H NMR spectrum and also by changes in the UV-Vis spectrum. The authors deduced a 1:1 stoichiometry of the complex from Job’s plot and found the value of the stability constant as 8.9 × 10^4^ M^−1^. A DFT study was performed to establish possible complexation modes. Finally, in vitro tests proved that while free betaine and **46** were not cytotoxic against MCF-7 and HeLa cell lines, the inclusion complex exhibited IC_50_ in the μg mL^−1^ range.

### 3.4. Self-Inclusion Complex of Calixarenes with Anticancer Drugs

**Figure 16:**
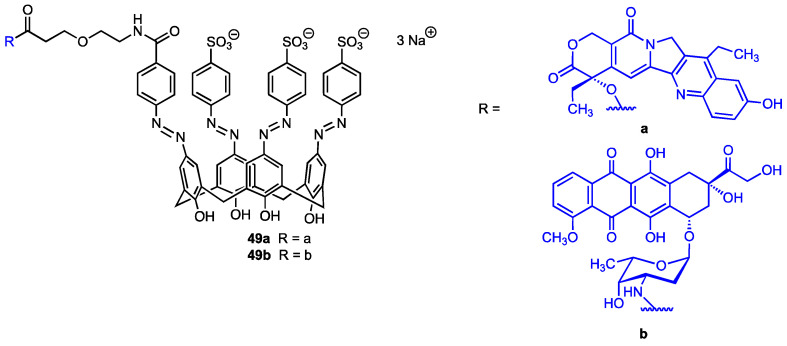
Calix[4]arenes bearing a 7-ethyl-10-hydroxycamptothecin (**49a**) or doxorubicin unit (**49b**).

In 2024, Cai, Guo, and co-workers [63] synthesised two calix[4]arene derivatives (Figure 16), each incorporating a single unit of chemotherapy drugs, 7-ethyl-10-hydroxycamptothecin (SN38) or doxorubicin (DOX), with the aim of preventing off-target release, thus reducing toxicity and side effects of these pharmaceuticals. The chemotherapeutic agents were covalently linked at the upper rim of the macrocycle through a suitably long chain containing an azo function, designed to be cleaved under hypoxia conditions, thereby leading to the release of SN38 or DOX at the tumour site. The formation of a self-inclusion complex (i.e., intramolecular host–guest conjugate), rather than intermolecular polymerisation, was confirmed by NMR spectroscopy and trapped ion mobility spectrometry−mass spectrometry (TIMS–MS). The anticancer performance of the two conjugates **49a** and **49b** was thoroughly evaluated in vitro using mouse breast cancer (4T1), human lung cancer (A549), and human breast cancer lines (MCF-7), as well as in vivo (mice). Moreover, additional in vivo studies were performed to evaluate the in vivo toxic and side effects in the mice (liver and kidney toxicity, cardiac tissue damage, loss of body weight, diarrhoea, and myelosuppression). The observed results demonstrated that the two self-inclusion complexes prevented premature drug leakage and mitigated some of the side effects associated with the 7-ethyl-10-hydroxycamptothecin and doxorubicin.

### 3.5. Calixarene-Based Micelles Encapsulating Anticancer Drugs

**Figure 17:**
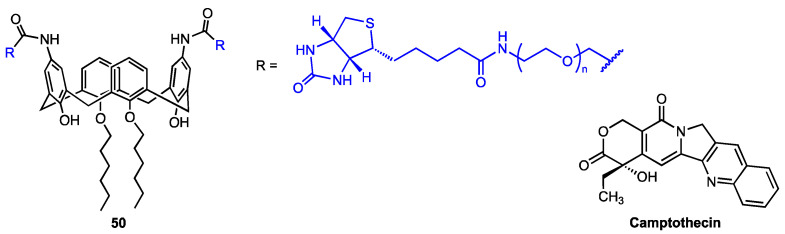
Structures of biotin-PEG-calix[4]arene and camptothecin.

Recently, An and co-workers [64] developed a supramolecular nanodelivery system for the highly toxic quinoline alkaloid camptothecin (CPT) based on the calix[4]arene derivative **50** (Figure 17), which features two biotin-PEG moieties at the upper rim (the actual length of the PEG chain was not known). The functionalised calixarene **50** exhibited a tendency to form micelles which efficiently encapsulated CPT, as proven by transmission electron microscopy and dynamic light scattering analyses. The drug release was found to be strongly pH-dependent. In vitro MTT assay revealed that CPT cytotoxicity against normal human endothelial cells (HUVEC) was significantly decreased by drug encapsulation (bringing it down to the level of the carrier itself, which was practically harmless). On the other hand, the IC_50_ value for CPT-**50** micelles (8.77 μM) was only slightly higher than that for the free camptothecin (8.03 μM) against the malignant cells (mouse breast cancer cells 4T1). The proapoptotic, concentration-dependent effect of the CPT-**50** micelles was further confirmed by annexin-FITC/PI staining.

## 4. Analogues of Calixarenes as Potential Anticancer Drugs

### 4.1. Dihomooxacalixarenes

**Figure 18:**
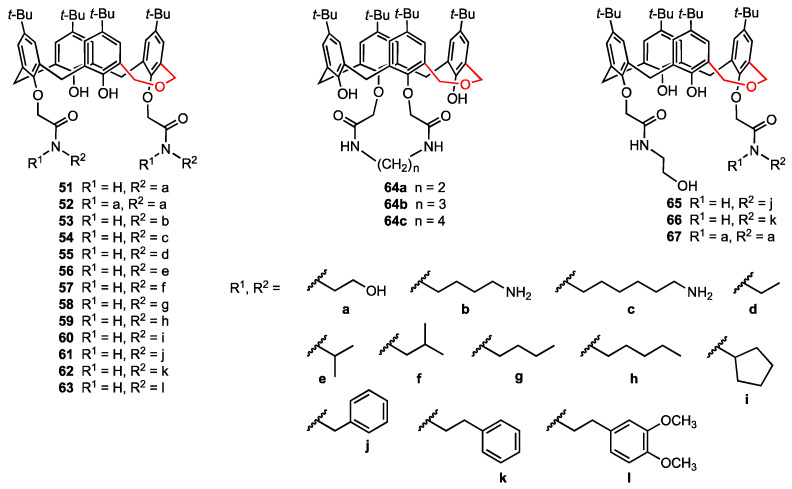
Functionalised dihomooxacalix[4]arenes.

In continuation of their work on the use of calixarene polyhydroxyamine derivatives as anticancer agents [38], An, Yan, and their co-workers [65] selected dihomooxacalix[4]arenes—more flexible analogues of calix[4]arenes where one methylene bridge is replaced by a CH_2_OCH_2_ unit [66,67]—as a new platform for the preparation of nineteen lower-rim bisamide derivatives, including three bridged macrocycles **64a**–**c** (Figure 18). The synthesised compounds were characterised by spectroscopic analysis, and the X-ray diffraction structures obtained for two of them confirmed their cone conformation. Cytotoxicity studies on A549, MCF-7, HeLa, and HepG2 cancer cell lines, as well as HUVEC normal cells, revealed that the compounds **51** and **54** were more potent (IC_50_ = 0.7–2.7 μM) than the previously reported calixarene bisamide **14** (see Figure 5). Moreover, flow cytometry analysis indicated that both compounds induce apoptosis of MCF-7 cells and cycle arrest in G0/G1 phase.

### 4.2. Thiacalixarenes

**Figure 19:**
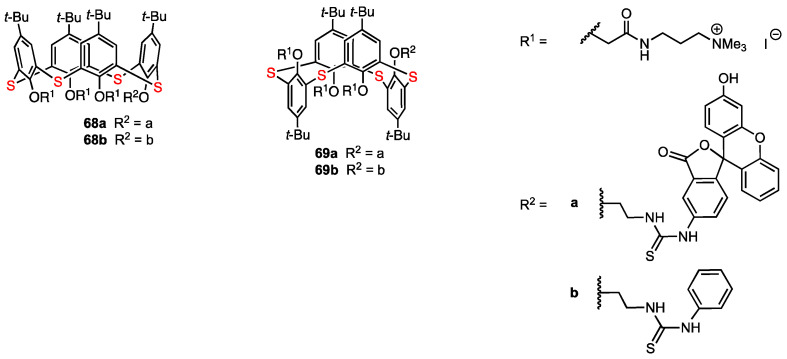
Functionalised thiacalix[4]arenes.

In 2022, Akhmedov, Stoikov, and their co-workers [68] reported the regioselective functionalisation of thiacalix[4]arene with a fluorescent label in order to obtain compounds with antiangiogenic activity that would act as galectin-1 inhibitors (Figure 19). These compounds were synthesised using as a reference the structure of anginex, a 33 amino acid peptide which is the most powerful galectin-1 inhibitor. In previous works, the hydrophilic and lipophilic fragments of anginex were taken into account, and some nonpeptidic surface topomimetics were synthesised by Dings and co-workers, including two calixarene-based compounds which proved to be potent angiogenesis inhibitors [69]. The whole series of functionalised macrocycles was then patented due to their antibacterial, antiangiogenic, and antitumour activity [70]. Akhmedov, Stoikov, and their co-workers envisaged the combination of the antiangiogenic drugs with diagnostic agents in order to obtain theranostic compounds. These researchers obtained thiacalix[4]arenes containing fluorescein fragments in both cone (**68a**) and 1,3-alternate (**69a**) blocked conformations. Additionally, the analogues containing a phenylthiourea moiety (**68b**, **69b**) were prepared to study the effect of the fluorescein unit on the cytotoxicity of the macrocycles.

The self-association of the synthesised compounds in aqueous solutions was studied. From the experimental data obtained for aggregates in water (DLS method), it appeared that the self-associates formed by 1,3-alternate macrocycles have a larger hydrodynamic diameter than those formed by the cone isomers. It was concluded that the macrocycles act as supramolecular self-associates when in solution, and they interact with cells in that form. A549 human lung adenocarcinoma cell line and HuTu-80 human duodenal adenocarcinoma cell line were used for the MTT test for cytotoxicity. The 1,3-alternate compounds **69a** and **69b** displayed no cytotoxicity against A549 cells over the entire range of applied concentrations, while the cone macrocycles **68a** and **68b** showed a cytotoxic effect on the cell line when the concentrations reached 50 μg/mL (**68a**) and 25 μg/mL (**68b**). Against HuTu-80 cells, **69a** was not cytotoxic at concentrations lower than 50 μg/mL, whereas **69b** and **68a** were cytotoxic at concentrations higher than 25 μg/mL. It was found that **68b** almost completely eliminated the cells at concentrations above 50 μg/mL. Therefore, the HuTu-80 cells were more sensitive to the cytotoxicity effect of the thiacalixarene derivatives; however, the 1,3-alternate isomers showed little or no toxicity, while the cone isomers were more cytotoxic. The presence of the fluorescein unit in the cone compound **68a** appeared to account for lower cytotoxicity levels when compared to the phenylthiourea derivative **68b**, which adopts the same blocked conformation. Next, the penetration of the thiacalixarene derivatives into living and dead A549 and HuTu-80 cells was evaluated using flow cytometry with propidium iodide. The penetrating ability of all compounds was found to be quite high for both living and dead cells. The newly obtained macrocycles could be promising theranostic compounds allowing the antiangiogenic treatment and monitoring the delivery using the fluorescent fragment. The lower cytotoxicity of the 1,3-alternate compounds compared to the cone isomers is consistent with the results of the previous work of Stoikov and co-workers [71]. Thus, differences in the mechanism of cell interaction between calixarene derivatives in 1,3-alternate and cone conformations are true not only in bacteria but also in human cells.

### 4.3. Calix[4]resorcinols

**Figure 20:**
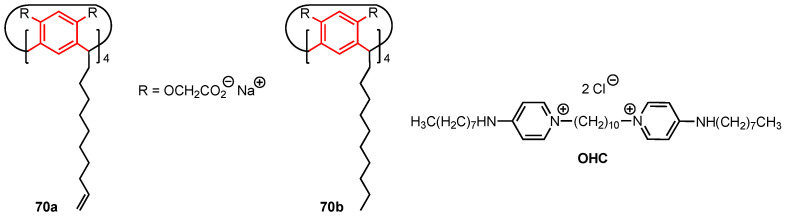
Structures of the calix[4]resorcinol derivatives and octenidine dihydrochloride (OHC).

In 2022, Kashapova and co-workers [72] explored the use of calix[4]resorcinol (calix[4]resorcinarene) scaffolds to develop self-aggregating systems incorporating octenidine dihydrochloride (OHC) as potential anticancer drugs. OHC is a commercial surfactant known to be an antimicrobial and anticancer agent; however, it is also highly toxic to healthy human cells. The two calix[4]resorcinol derivatives **70a** and **70b** (Figure 20), functionalised with eight negatively charged acetate groups at the upper rim and lipophilic chains at the lower rim, were mixed with octenidine dihydrochloride to obtain spherical nanoparticles (~100 nm by TEM microscopy) which were expected to display higher selectivity toward cancer cells compared to the standalone surfactant. The cytotoxicity of **70a**, **70b**, OHC, OHC–**70a**, and OHC–**70b** (with 6:1 and 12:1 OHC to calix[4]resorcinol ratios used to form the aggregates) was evaluated against the MTT (human cervical carcinoma), HuTu (human duodenal adenocarcinoma), MCF-7, and A549 cancer cell lines, as well as the healthy Chang liver cell line. The calix[4]resorcinol derivatives **70a** and **70b** did not display any significant anticancer activity (IC_50_ > 50 µM) and did not show cytotoxicity against the healthy human cells. On the other hand, the aggregates OHC–**70a** and OHC–**70b** displayed high cytotoxicity against the healthy human cell line (IC_50_ = 1.9 µM for 12:1 OHC–**70a**). However, these aggregates displayed reduced cytotoxicity against the MTT and A549 cancer cell lines, raising concerns about their effectiveness against cervical and lung cancers. The results for the MCF-7 and HuTu 80 cell lines proved to be more promising, with the aggregates reaching IC_50_ values of 0.4 µM against the latter and 1.2 µM against the former cell line. The most interesting system appeared to be the OHC-**70b** aggregate at a 6:1 ratio, which showed high anticancer activity (IC_50_ = 0.4 µM against the HuTu and 1.9 µM against the MCF-7 cell line). Its cytotoxicity against the healthy cells, while lower than its 12:1 counterpart, was still relatively high at 8.1 µM. Similarly, the OHC-**70a** aggregate at a 6:1 ratio displayed cytotoxicity of 9.3 µM against the healthy cells, 0.4 µM for the HuTu cell line, and 5.2 µM for the MCF-7 cell line. This research indicates that the amphiphilic character of the aggregates improves the selectivity of OHC against cancer cells, with the anticancer activity against human duodenal adenocarcinoma especially worth further research.

**Figure 21:**
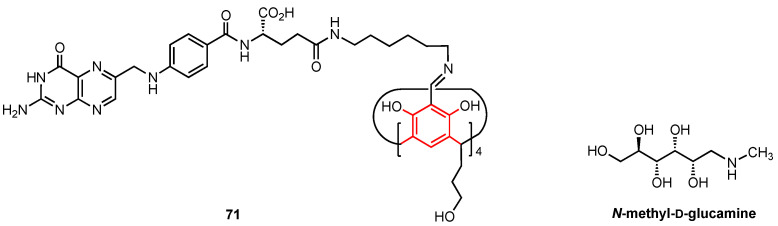
Calix[4]resorcinol bearing four folic acid moieties.

One year later, the same research team described the synthesis of the folic acid-containing calix[4]resorcinol derivative **71** (Figure 21), designed to selectively target cancer cells through interaction with the folic acid alpha receptors which are overexpressed in some tumours [73]. Since **71** was not water-soluble, the addition of a 220-fold excess of *N*-methyl-D-glucamine was necessary to increase its solubility (through a hydrotropic effect) and thus allow the complexation of the anticancer drug doxorubicin. The activity of **71** and its doxorubicin complex was analysed using M-HeLa cells and human hepatocyte cells (Chang liver cell line). A similar cytotoxic effect on both the cancerous and healthy cells (IC_50_ = 17.0 and 20.0 µM, respectively) was observed for **71**, while the water-soluble complex was more active, with IC_50_ values of 2.0 µM (HeLa cell line) and 5.0 µM (Chang liver cell line) being found, lower than those observed for pure doxorubicin (IC_50_ = 5.0 and 6.0 µM, respectively). It is worth noting that the *N*-methyl-D-glucamine itself did not show any cytotoxicity against both cell lines. The relatively low selectivity displayed by the complex suggests that further investigation is needed to obtain a compound that could be successfully used in cancer treatments while providing high selectivity towards malignant cells over healthy cells.

### 4.4. Azacalixarenes

**Figure 22:**
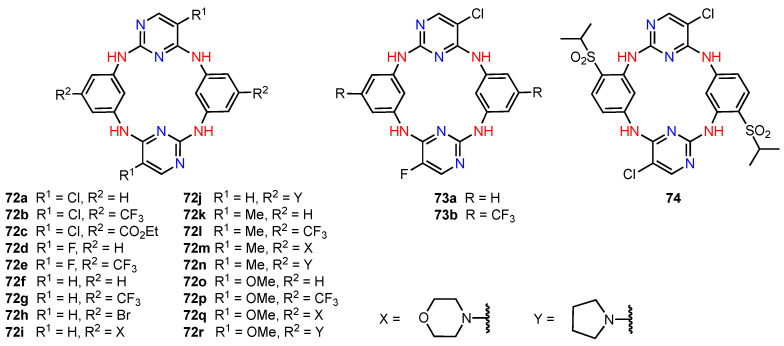
Anticancer azacalix[2]arene[2]pyrimidine derivatives.

Aiming at combining the versatile calixarene skeleton with the well-known biological activity of 2,4-diaminopyrimidines, Wang, He, and their co-workers designed a series of azacalix[2]arene[2]pyrimidines (**72a**–**r**, **73a**,**b** and **74**, Figure 22), in which two phenyl and two pyrimidine fragments were joined through -NH- bridges [74]. The target compounds were prepared in two steps from appropriately substituted 2,4-dichloropyrimidines and benzene 1,3-diamines, and their structures were confirmed by NMR spectroscopy, mass spectrometry, and, only for the dichloro derivative **72a**, by X-ray diffraction analysis. The twenty-one new azacalix[2]arene[2]pyrimidines were subjected to the CCK-8 colorimetric assay to evaluate their anticancer potential. While most compounds exhibited little to no activity, six products (**72g**, **72h**, **72j**, **72k**, **72n**, **72r**) showed significant cytotoxicity (IC_50_ = 0.58–18.75 μM) for A549 (human lung adenocarcinoma), MCF7 (human breast cancer), and Sy5Y (human neuroblastoma) lines, and lower cytotoxicity (IC_50_ = 15.6–46.5 μM) for CNE cells (human nasopharyngeal carcinoma). Notably, the most active pyrrolidine-substituted azacalix[2]arene[2]pyrimidine **72j** featured inhibitory properties comparable to those of the benchmark drug doxorubicin while being considerably less toxic for the normal human foetal hepatocyte line L-02. Additional tests on the MCF7 cell line shed light on the mechanism of action of this compound, where cell apoptosis through induction of the caspase-3–caspase-9 signalling pathway was detected.

### 4.5. Calix[4]pyrroles

**Figure 23:**
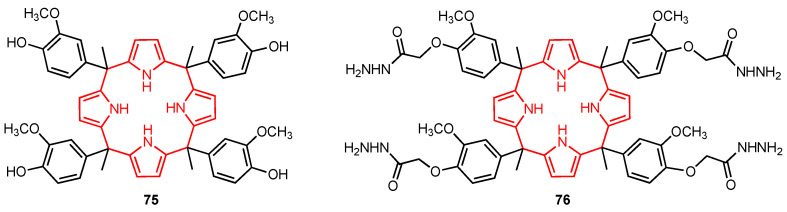
Anticancer calixpyrrole derivatives.

Calix[4]pyrroles have a wide range of applications, including ion recognition and biomedical applications [75], and their cytotoxic activity against certain cancer cell lines has also been reported in the past. In 2022, Kongor, Bhatt, and their co-workers [76] synthesised the calix[4]pyrrole derivatives **75** and **76** (Figure 23) and evaluated their cytotoxicity on HeLa and MCF-7 human cancer cell lines using adriamycin as a positive control. The study showed that **75** had good anticancer activity against HeLa and MCF-7 human cancer cell lines, displaying higher potency than **76**. The assays revealed that **75** was very active at concentrations of 10–80 μg/mL. The authors suggested that this behaviour was due to the fact that **75** had more hydrogen bond donor functional groups compared to **76** (with phenolic hydroxyl groups playing the most important role). Although the presence of the OH groups might be the reason for the higher anticancer efficacy of **75**, the specific mechanism of action is still unclear. Moreover, since molecular modelling studies showed that **75** can penetrate the quinone reductase-2 pocket, this molecule could be further explored as a potential anticancer drug.

### 4.6. Pillar[5]arenes

Pillar[5]arenes, first reported in 2008, are macrocycles constituted of 1,4-disubstituted hydroquinones linked by methylene bridges in the 2,5-positions [77,78]. They feature a more rigid structure than calixarenes, and their modification is often more convenient when compared to other cyclooligomers, e.g., cyclodextrins. Recently, they have been utilised for the synthesis of supramolecular polymers endowed with anticancer properties [79].

**Figure 24:**
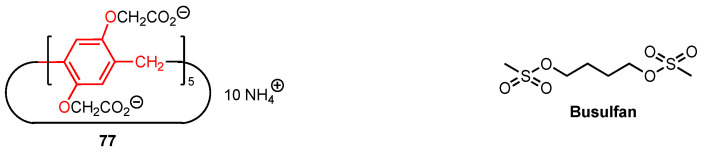
Structures of carboxypillar[5]arene and anticancer drug busulfan.

In 2022, Dong, Zhang, and their co-workers [80] discovered that the negatively charged host, carboxypillar[5]arene **77** (Figure 24), was able to encapsulate the anticancer drug busulfan leading to reduced hydrolytic degradation and a significant increase in its water solubility. In vitro studies of the complex against hepatocellular carcinoma cells (HepG-2) proved that the cytotoxicity of the drug was essentially maintained after four days (IC_50_ = 404.6 μM versus 333.5 μM for fresh busulfan and 719.5 μM after four days in solution without the macrocyclic host **77**).

**Figure 25:**
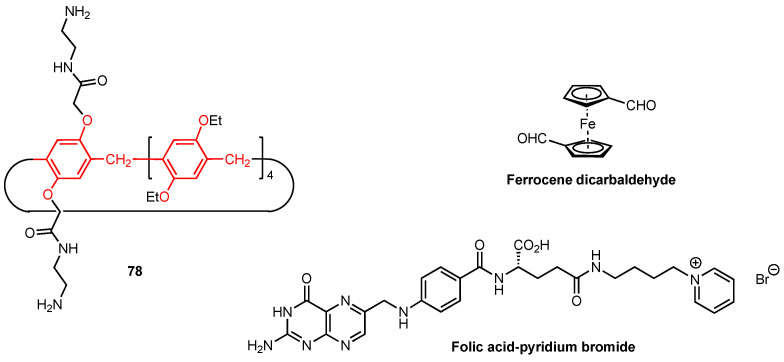
Structures of amino-functionalised pillar[5]arene, ferrocene dicarbaldehyde, and folic acid-pyridinium bromide salt.

The same year, Wang and co-workers [81] synthesised supramolecular nano-platforms (NPs) based on the imino-linked polymers formed by the bis-amine pillar[5]arene **78** and the ferrocene dicarbaldehyde monomers (Figure 25). Glucose oxidase (GOx) was employed to assist intermolecular H-bonding, and the resulting nanoplatforms were used to host the anticancer drug doxorubicin (DOX) as well as the folic acid-pyridinium bromide salt (FA-Py) (on the NPs surface) to target the cancer cells. The antitumour activity of the supramolecular systems was first evaluated in vitro using HeLa cancer cell line. The NPs hosting both DOX and FA-Py exhibited a strong targeting effect on cancer cells, with cell viability reaching 6% for a concentration of 120 µg/mL. At the same time, the cell viability remained high for the dynamic covalent polymers alone (80% viability at 120 µg/mL), suggesting that the doxorubicin is effectively released in the tumour cell environment due to its low pH. The antitumour activity was then tested in vivo, using mice with a tumour volume of around 100 mm^3^ that were injected with the various nanoplatforms every three days. The body weight of the mice was monitored throughout the experiment and was determined to be constant, indicating that the nanoplatforms are safe and cause relatively insignificant initial side effects. The tumour volume was also monitored, and while it increased for the control group, as well as for most of the groups treated with the simple imino-linked pillar[5]arene-ferrocene polymers, the GOx/DOX/Fa-Py nanoplatforms showed an inhibitory effect on the tumour size, with a reduction in tumour cell count during the experiment. Additional staining of vital organs (heart, liver, spleen, lungs, and kidneys) revealed no signs of inflammation or tissue damage, further supporting the safety of these nanoplatforms.

The mechanism of the drug delivery is based on the folic acid conjugated with a pyridinium group, which targets the cancer cells by binding to the folate receptors on their surface, allowing the nanoplatforms to accumulate in the tumour tissue. Then, GOx catalyses the oxidation of glucose, which is typically abundant in cancer cells due to their high metabolic rate, producing hydrogen peroxide as a byproduct. The ferrocene unit within the nanoplatform then catalyses the decomposition of H₂O₂ to generate hydroxyl radicals that induce oxidative damage to cellular components, leading to cell death (chemodynamic therapy, CDT). In addition to facilitating CDT, the nanoparticles also carry the chemotherapeutic drug doxorubicin (DOX). The acidic conditions in tumour tissues trigger the release of DOX, which then exerts its cytotoxic effects on the cancer cells, damaging their DNA and leading to apoptosis. The combination of CDT (via hydroxyl radicals) and chemotherapy (via DOX) provides a synergistic antitumour effect. This work not only proposed a new supramolecular drug delivery platform but also provided interesting in vivo results, which are notable given the rarity of such consistently favourable outcomes in similar studies. These nanoplatforms deserve further research and development, considering their promising antitumour activity and the lack of notable side effects after the in vivo treatment.

## 5. Conclusions

The previous reviews on this topic [19,20,21,22,23,24,25] and the recent works reviewed in the present paper indicate that the development of new calixarene-based (and calixarene analogues) anticancer agents is a quite active research field. Nevertheless, the pharmacological properties of these compounds were assayed using human cell lines, while in vivo studies were rarely performed. In fact, only the calixarene derivatives **36** (Figure 10), hosting the chemotherapeutic drug methotrexate, **49a**,**b** (Figure 16) bearing the 7-ethyl-10-hydroxycamptothecin or doxorubicin anticancer agents, and the supramolecular nanoplatform based on the pillar[5]ene **78** (Figure 25) were submitted to in vivo (mice only) assays. The reason for the lack of in vivo tests may be related to the quite poor water solubility of the calixarenes, especially those larger than the most common member of this class, the calix[4]arene, even when they carry hydrophilic substituents (e.g., alcohol, amine, amide, and urea groups). Often, these macrocycles require ionised substituents like the quaternary ammonium cations or the sulfonate anions to become water-soluble without the addition of cosolvents. Further synthetic efforts are thus needed to obtain novel drug candidates constituted of functionalised calixarenes or calixarene analogues featuring suitable pharmacokinetic behaviours. Moreover, most of the products synthesised during the last years are not homochiral, the exceptions being the calixarenes covalently linked to the amino acids L-proline (**2**–**6**, Figure 2; **7a**,**b** and **8a**,**b**, Figure 3), L- or D-aspartic acid (**9** and **11**, Figure 4), and L- or D-glutamic acid (**10** and **12**, Figure 4), to enantiopure drugs **49a**,**b** (Figure 16), or to the biotin (**50**, Figure 17). Among the analogues of calixarenes, only the calix[4]resorcinol linked to four folic acid units (**71**, Figure 21) was a homochiral molecule. It appears that the chirality issue was scarcely investigated; therefore, more research in this field should lead to new compounds endowed with more selective anticancer activity.

## Data Availability

No new data were created or analyzed in this study.
